# Use of a Slow-Release Phenylalanine-Free Microtablet Protein Substitute in Children and Adolescents with Phenylketonuria: An Observational Pilot Study

**DOI:** 10.3390/nu18142375

**Published:** 2026-07-21

**Authors:** Martina Tosi, Anne Daly, Catherine Ashmore, Ozlem Yilmaz Nas, Alex Pinto, Sharon Evans, Anita MacDonald

**Affiliations:** Department of Dietetics, Birmingham Women’s and Children’s Hospital, Birmingham B4 6NH, UK; a.daly3@nhs.net (A.D.); catherine.ashmore@nhs.net (C.A.); ozlem.yilmaz@nhs.net (O.Y.N.); alex.pinto@nhs.net (A.P.); sharon.morris6@nhs.net (S.E.); anita.macdonald@nhs.net (A.M.)

**Keywords:** phenylketonuria, phenylalanine, protein substitutes, slow-release, microtablets, acceptability, tolerance, gastrointestinal symptoms

## Abstract

**Background/Objectives**: In phenylketonuria (PKU), adherence to protein substitute (PS) is frequently suboptimal due to poor palatability, high volume, and sensory fatigue. A novel phenylalanine (Phe)-free L-amino acid microtablet PS has been developed to address these barriers. The microtablets incorporate a cellulose-based taste-masking coating and a sodium-alginate inner matrix intended to enable controlled amino acid release over approximately three hours. This study evaluated their short-term tolerability, acceptability, and adherence in children with PKU, with extended follow-up in a subgroup. **Methods**: A 7-day observational study was conducted in participants with PKU aged ≥3 years on a low-Phe diet in a single centre. The test product provided protein equivalent (PE) 54 g/100 g. Participants replaced at least one daily dose of their usual PS with the test product, providing 10 g or 20 g/day PE, with one child receiving 80 g/day. Daily gastrointestinal (GI) symptoms, PS intake, and adherence were recorded. Acceptability was assessed using structured ratings of palatability, ease of use, and overall preference. Longer-term tolerability and acceptability were then assessed in four adolescents who elected to continue the test product for an additional 28 days. **Results**: Ten participants completed the 7-day study. Adherence to the test product was high (80%), exceeding adherence to participants’ usual PS (70%). At baseline, nine participants reported none/mild GI symptoms, most commonly flatulence. One participant reported moderate/severe symptoms including constipation, flatulence, diarrhoea, bloating, burping, and abdominal discomfort. By day 7, all participants reported no or mild symptoms, with complete resolution in the child with moderate/severe baseline symptoms. Acceptability ratings were comparable between the test product and usual PS, and nine children (90%) reported no difficulty taking the test product. During the 28-day extension, adherence remained high, GI tolerance was maintained, and improved preference was noted, particularly due to reduced aftertaste. **Conclusions**: This novel slow-release PS microtablet was well tolerated, acceptable, and associated with high adherence. Its taste-masked, low-volume format offered practical advantages that may support sustained dietary adherence. Longer-term controlled studies are necessary to confirm these findings.

## 1. Introduction

Phenylketonuria (PKU, OMIM 261600) is an autosomal recessive metabolic disorder caused by pathogenic mutations in the phenylalanine hydroxylase (PAH) gene, resulting in reduced or absent enzyme activity and consequently elevated blood phenylalanine (Phe) levels [1,2]. Treatment is based on a lifelong low-Phe diet, which involves a carefully controlled and restricted intake of natural dietary protein to meet essential Phe requirements [3,4]. The majority of protein needs are provided through Phe-free or low-Phe protein substitutes (PS) [5], supplying all amino acids except Phe and contributing additional tyrosine (Tyr) [6]. In classical PKU, PS may account for up to 70–80% of total nitrogen intake and they typically include varying amounts of carbohydrates, fat, long-chain fatty acids and may contain vitamins, and minerals [7].

Adherence to both the low-Phe diet and PS usually declines progressively with age and is closely linked to worsening blood Phe control [8]. In PKU, subtle neurocognitive, psychological, behavioural, and emotional difficulties, frequently observed across childhood, adolescence, and adulthood, may further erode long-term engagement with dietary treatment [9]. Across studies, the unpleasant taste, odour, and lingering aftertaste of synthetic free L-amino acid-based PS are consistently identified as important barriers to sustained adherence [10]. Although advances in formulation technology have sought to improve palatability, through odour masking, bitterness reduction, and lowering the daily volume burden, sustaining adequate, regular intake remains particularly challenging in paediatric populations, where sensory sensitivities, feeding behaviours, and daily routines can amplify aversion and variability in consumption [11,12].

Several formulations of PS are available for the dietary management of PKU, including free L-amino acid-based PS, casein glycomacropeptide supplemented with amino acids (CGMP-AA), and slow- or prolonged-release formulations. Synthetic L-amino acid-based PS bypasses proteolytic digestion, leading to a rapid rise in circulating amino acids, characterized by higher postprandial peaks and faster declines than those observed with intact dietary proteins [13]. Consequently, clinical guidance typically recommends that PS be consumed in small, evenly distributed doses throughout the day to support more stable amino-acid availability [14]. When intake is infrequent or irregular, marked fluctuations in plasma amino acid concentrations may occur over a 24-h period, a pattern associated with poorer metabolic control in individuals with PKU [15].

Modified-release formulations have been developed to more closely mimic physiological protein digestion, with the aim of improving amino acid utilisation and reducing post-prandial variability in plasma concentrations [15]. The rapid absorption of free L-amino acids is associated with increased amino acid oxidation, greater protein catabolism, and higher urinary nitrogen excretion, highlighting the potential metabolic advantages of modified-release technologies [14,16]. In addition to these physiological benefits, modified-release formulations may improve organoleptic characteristics, enhancing palatability and acceptability, which may ultimately contribute to improved long-term adherence to dietary treatment in PKU [17].

**Figure 1:**
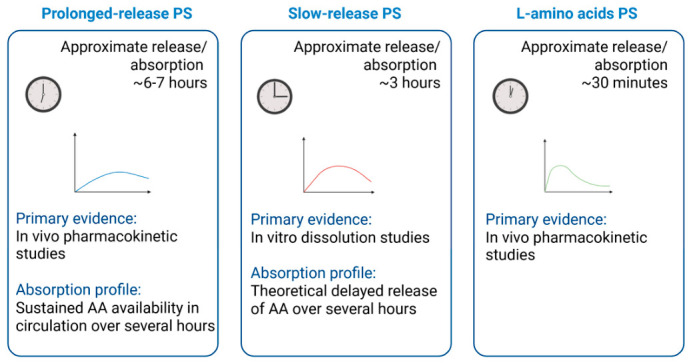
Differences in dissolution and kinetic assessment of different amino acid PS. Prolonged-release formulations and L-amino acid PS have been evaluated using pharmacokinetic measures (e.g. Time to Maximum concentration (Tmax), Peak plasma concentration (Cmax), Area Under the Curve (AUC) and duration of elevated plasma amino acid concentrations) [15,16,18,19], whereas information on slow-release formulations is primarily derived from manufacturers’ technical data describing in vitro release parameters (e.g., percentage amino acid release over time and Time to 50% release/dissolution). Abbreviations: PS = Protein Substitutes; AA = Amino Acid.

Within modified-release PS, a distinction should be made between prolonged-release and slow-release formulations. Prolonged-release PS are supported by formal kinetic studies demonstrating sustained plasma amino acid concentrations over approximately 6–7 h, indicating a controlled and extended absorption profile that promotes greater stability in plasma amino acid levels [15,16,18,19]. In contrast, slow-release formulations, primarily characterised using in vitro dissolution data, demonstrate delayed amino acid release over approximately 3 h but lack formal in vivo kinetic characterisation. This differs markedly from L-amino acid-based PS, which are rapidly absorbed and typically reach peak plasma concentrations within around 30 min of ingestion [14]. These differences in dissolution behaviour and the strength of kinetic evidence reflect distinct absorption profiles across formulations, which are directly relevant to their differential use in the dietary management of PKU. The main differences between the PS are summarised in Figure 1.

The aim of this observational study was to evaluate the efficacy, acceptability, and tolerability of a newly developed slow-release, Phe-free microtablet PS, fortified with vitamins and minerals, in individuals with PKU aged 3 years and older.

## 2. Materials and Methods

### 2.1. Study Design

This prospective observational pilot study evaluated the acceptability of a novel slow-release, Phe-free microtablet PS in children with PKU aged 3 years and older, all of whom were followed at a single specialist metabolic centre in the United Kingdom. The study protocol comprised a 7-day, short-term intervention conducted in the home environment, with the primary objective of assessing product acceptability and gastrointestinal (GI) tolerance under real-world conditions.

### 2.2. Inclusion and Exclusion Criteria

Children and adolescents aged 3 years or older with a confirmed diagnosis of PKU through newborn screening were eligible for inclusion. Participants were required to have commenced a Phe-restricted diet from the time of diagnosis, to be adherent to a prescribed Phe-restricted diet, and to use PS regularly. Both the child and caregiver were required to demonstrate the ability and willingness to complete the 7-day study procedures.

Exclusion criteria included age under 3 years, the presence of significant comorbidities, poor adherence to dietary management, participation in another clinical trial within the preceding two weeks, antibiotic use within the two weeks prior to enrolment, and any concerns raised by the lead dietitian regarding the participant’s capacity or motivation to comply with the study protocol.

### 2.3. Study Product

The study product, PKU EASY Microtabs Plus (POA Pharma/Galen, Malmö, Sweden), was a Phe-free amino acid based PS formulated as microtablets for children aged ≥3 years with PKU. It contained a blend of essential and non-essential amino acids, together with vitamins, minerals, and trace elements. Each microtablet was small, approximately 3 × 4 mm in size. The formulation used sodium alginate as a hydrophilic carrier, which was wet-granulated with an aqueous amino acid solution, dried, and subsequently coated with hydroxypropyl methylcellulose and a protective film to produce small cylindrical microtablets. This controlled-release matrix was designed to modulate the liberation of amino acids over approximately 3 h.

The product was administered using a dosing cup, with the 25-mL fill line delivering 10 g of protein equivalent (PE), corresponding to 20 g of product and providing 68 kcal (Table 1).

### 2.4. Study Procedures

Each participant received a home visit from a research dietitian, during which the study product was introduced and individualised guidance on dosage and administration was provided. Caregivers and children were instructed to replace at least one daily serving of their usual PS with the study product, corresponding to an intake of 10–20 g/day of PE.

The study product was administered with water or an aspartame-free flavoured squash. During its first administration, children were supervised and advised to consume the study product while seated upright. Participants’ usual PS were either a combination of low-Phe CGMP-AA based formulations with Phe-free amino acid-based PS or Phe-free amino acid–based PS.

### 2.5. Assessments

Subject demographics were recorded at study baseline.

#### 2.5.1. Dietary Assessment and Anthropometry

Dietary intake and anthropometric parameters, including body weight, height, and BMI z-score, were assessed at baseline and on day 7. Measurements were performed by two trained research dietitians (AM, AD). Height was recorded to the nearest 0.1 cm using a Seca stadiometer, and body weight to the nearest 0.1 kg using calibrated digital scales (Seca Medical Measuring Systems and Scales, Birmingham, UK; Model 875). Medical history, concomitant medications, and any treatment-related adverse reactions were systematically documented.

Participants were instructed to maintain their usual low-Phe diet throughout the study, so total PE intake was stable. Dietary intake was assessed using structured 3-day food diaries, completed by caregivers. Baseline dietary data included Phe intake, PS consumption, and total protein intake prior to the intervention. All dietary records were subsequently reviewed and analysed by trained metabolic dietitians.

Adherence to the study product was monitored using a structured daily diary completed by caregivers during the 7-day intervention. Adherence was defined as the extent to which the study product was consumed according to the prescribed dose and frequency, with daily intake recorded quantitatively.

#### 2.5.2. Metabolic Monitoring

In addition to samples collected during the study, three retrospective dried blood spot (DBS) measurements were available for each participant. Caregivers collected fasting DBS samples at home both prior to study initiation and at the end of the 7-day intervention. Target blood Phe concentrations were defined according to age: 120–360 µmol/L for children aged ≤12 years and 120–600 µmol/L for those >12 years [1].

As part of routine clinical practice, early-morning fasting dried blood spot (DBS) samples were collected using standardised filter paper cards (PerkinElmer 226; UK Standard NBS, Public Health England, London, UK). Specimens were posted to the Birmingham Children’s Hospital laboratory for analysis. All samples were processed using uniform card thickness, and Phe and Tyr concentrations were quantified from a 3.2-mm punch by tandem mass spectrometry (Waters Xevo TQD, Elstree, UK). No correction factor was applied to convert DBS concentrations to plasma values, as all measurements were performed consistently using the same validated DBS analytical method throughout the study.

DBS Phe and Tyr concentrations were available for all participants at baseline (T0) and after the 7-day intervention (T1). Median blood Phe and Tyr concentrations at T0 (baseline) were calculated using the mean of three fasting DBS samples per participant. Post-intervention values were obtained from a single fasting DBS sample. Results are reported as median values for the cohort only.

#### 2.5.3. Gastrointestinal Tolerance Assessment 

GI tolerance was assessed at baseline and on day 7 using a structured symptom checklist completed by caregivers and/or participants. The tool assessed for diarrhoea, constipation, bloating or abdominal distension, nausea, vomiting, burping or regurgitation, flatulence, and abdominal pain or discomfort. Symptom severity was graded using a four-point ordinal scale (none, mild, moderate, severe). Throughout the 7-day intervention, caregivers maintained a daily diary to document any changes in GI symptoms.

#### 2.5.4. Acceptability Assessment

At baseline and on day 7, participants and their caregivers completed a structured, non-validated questionnaire designed to compare the novel, Phe-free product with the participant’s usual PS. The assessment captured key sensory and practical attributes, including appearance, odour, taste, texture, ease of preparation, ease of consumption, and aftertaste. Responses were recorded using a five-point scale ranging from *Great* (0) to *Terrible* (4), with an additional option of *Not applicable*. Scores were subsequently converted into numerical values for analysis.

### 2.6. Follow-Up Study

A subset of adolescent participants continued using the study PS for an additional 28 days beyond the 7-day intervention period, with data collection extended accordingly. During this extended period, anthropometric measurements, GI tolerance, adherence, and DBS Phe and Tyr concentrations were monitored. In participants who continued long-term use of the study PS, observational follow-up data up to 6 months post-intervention were obtained from routine clinical records, including all available DBS Phe measurements collected during standard clinical care.

### 2.7. Ethical Approval

Ethical approval for this study was obtained from the Health Research Authority (HRA) and Health and Care Research Wales (HCRW) and the East of England. Cambridge South Research Ethics Committee (IRAS Project ID: 320040, approved on 8 October 2024). Caregivers of eligible participants received detailed study information sheets, and all procedures were explained by trained research dietitians. Written informed consent was obtained from caregivers prior to enrolment in the 7-day study, and assent was obtained from children where appropriate. Participants were informed of their right to withdraw from the study at any time during the intervention period. The study was conducted in accordance with the Declaration of Helsinki.

### 2.8. Statistical Analysis

No formal sample size calculation was performed, as all eligible participants meeting the inclusion criteria were recruited. Given the small sample size, no inferential statistical analyses were conducted, and results are presented using descriptive statistics only. Continuous variables are reported as mean or median, ordinal variables are summarised using medians, while categorical variables are expressed as counts and percentages.

## 3. Results

### 3.1. Subjects

Ten children with PKU were enrolled and completed the 7-day study by July 2025. The cohort included five males and five females, with a median age of 14.5 years (range: 5.1–17.5 years). Four participants were White-British, four were White-other, and two were of British Pakistani ethnicity. Baseline demographic characteristics are presented in Supplementary Table S1.

### 3.2. Anthropometric Measurements

Weight, height, and BMI z-scores remained stable over the study, and were calculated according to the WHO Growth Reference for children and adolescents aged 5–19 years [20]. Supplementary Table S2 describes the anthropometry.

### 3.3. Dietary Assessment, Protein-Substitute Intake and Adherence to the New PS

Two children (participants 6 and 10) were receiving sapropterin (20 mg/kg) as adjunctive therapy; the remaining participants had sapropterin-non-responsive genetic variants. At baseline, most children (*n* = 9/10) were prescribed an amino-acid–based PS. One participant used a combination of a powder CGMP-AA based PS and a ready-to-drink amino-acid (RTD-AA) based PS.

Across the cohort, RTD-AA based PS was the most frequently used (*n* = 7/10), followed by powders (*n* = 2/10) and tablets or microtablets (*n* = 2/10). One participant used two PS. The prescribed dosing frequency ranged from three to four servings per day. The median PE intake from PS was 80 g/day (range: 60–80 g/day) (Table 2).

Natural protein prescriptions, expressed as protein exchanges (1 g protein exchange ≈ 50 mg Phe), were below 10 protein exchanges/day in 8 of 10 participants, whereas the remaining two participants were prescribed 13 and 20 protein exchanges/day (Table 2).

During the intervention period, the study PS was prescribed at doses corresponding to 10–20 g/day of PE for most participants, with one child receiving 80 g/day. One participant supplemented, rather than replaced, their usual PS by adding 10 g/day of PE from the new PS. The majority were instructed to take the product once daily, while one participant received four divided doses (Table 2). The microtablets were administered with water in all cases.

All participants (*n* = 10) completed the 7-day intervention. Adherence to the study product was high, with 8 of 10 children (80%) reporting full adherence and 2 of 10 reporting partial (“mostly taken”). For their usual PS, 7 of 10 participants (70%) reported full adherence (Table 2).

### 3.4. Metabolic Control

Median blood Phe concentrations at T0 were 465 µmol/L (range: 120–770 µmol/L), based on the mean of three fasting DBS samples per participant. After the 7-day intervention (T1), the median Phe concentration was 460 µmol/L (range: 80–1190 µmol/L). Median Tyr concentrations at T0 were 63 µmol/L (range: 30–190 µmol/L), and 65 µmol/L (range: 40–130 µmol/L) at T1.

### 3.5. Gastrointestinal Tolerance

**Figure 2:**
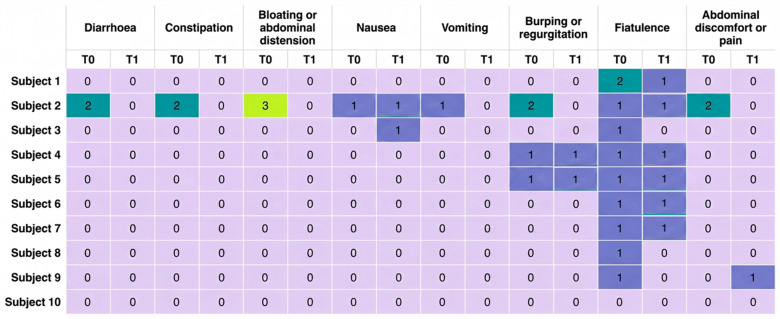
Gastrointestinal tolerance and symptoms assessed at T0 and at T1. Symptom severity was scored as follows: 0 = none (lavender), 1 = mild (purple), 2 = moderate (green), and 3 = severe (yellow).

At baseline, 9 of 10 participants (90%) reported no or only mild GI symptoms while using their usual PS. Flatulence was the most frequently reported symptom (*n* = 9/10; 90%), with one participant rating it as moderate. One child (participant 2) reported moderate-to-severe GI symptoms, including diarrhoea, constipation, burping, and abdominal discomfort (all rated as moderate), and bloating/abdominal distension (rated as severe) (Figure 2).

By the end of the 7-day intervention, all participants (*n* = 10/10; 100%) reported no or mild GI symptoms. Participant 2 experienced complete resolution of severe and moderate symptoms following introduction of the study PS (Figure 1). Participant 2 was receiving an osmotic stimulant laxative at both T0 and T1; no other participants were taking medication.

### 3.6. Acceptability

Participants rated both their usual PS and the study product across a range of organoleptic and usability characteristics. Ratings were recorded on a five-point scale, with lower scores indicating better acceptability. Median scores for each criterion are presented in Table 3. Overall, there were no meaningful differences between the usual PS and the study product.

Most children (*n* = 8/10) reported no difficulties consuming the study product during the 7-day intervention. One participant initially experienced difficulty but adapted to the microtablet formulation with practice, while another participant continued to report challenges. For participant 8, who used two different baseline PS (a RTD product and a powdered formulation), mean scores across the two products were calculated for comparison.

No adverse events were reported.

### 3.7. Follow-Up Study

Four adolescents (participants 2, 6, 8, and 10) continued using the study PS for an additional 28-day follow-up period. Participants 2, 6 and 10 received their entire daily PS intake from the study product, corresponding to 80 g, 70 g and 80 g PE/day, respectively. Participants 2 and 6 consumed the PS in four divided doses (8 × 25-mL cups/day in total and 7 × 25-mL cups/day in total, respectively). Participant 10 consumed 50 mL per dose, four times daily (8 × 25-mL cups/day in total), whereas participant 8 consumed 20 g PE/day from the study product (2 × 25-mL cups/day), representing approximately 25% of his usual daily intake.

Growth parameters remained stable, with all adolescents maintaining their respective height and weight z-scores throughout the extended follow-up. Mean Phe and Tyr were calculated for each participant using all available DBS samples collected during the 28-day period (Table 4). Adherence was consistently good across all participants, except for participant 2, where it was described as suboptimal. No GI intolerance was reported during the extended use of the study product.

Three adolescent participants (participants 6, 8, and 10) elected to continue long-term use of the study PS. Long-term metabolic control was evaluated in these individuals during a 6-month post-intervention period. All available DBS measurements collected during this timeframe, including those obtained during the initial 28-day follow-up, were included in the analysis.

For each participant, the total number of DBS measurements and the proportion within the target Phe range (120–600 µmol/L) were calculated. Participant 6 had 13 DBS measurements, of which 10 (76.9%) were within the target range. Participant 8 had 20 measurements, with 19 (95.0%) within range. Participant 10 had 9 measurements, of which 7 (77.8%) were within the recommended range.

## 4. Discussion

In this study, the introduction of a novel slow-release microtablet PS, with added vitamins, minerals, and trace minerals in children and adolescents with PKU was associated with high adherence and sustained long-term use in a subset of children and adolescents with PKU. Despite the relatively high number of microtablets required each day, most participants (*n* = 9/10) were able to consume the product successfully during the short-term intervention, although the cohort was predominantly adolescents. The product was well tolerated. During follow-up, several participants increased their intake of the study product, suggesting progressive acceptance and behavioural adaptation with continued exposure. Notably, one participant transitioned completely from their previous PS to the study product within the initial 7-day period. Metabolic control remained stable throughout both the short-term intervention and the extended follow-up, with no deterioration in Phe concentrations.

The current evidence base for Phe-free slow- and prolonged-release PS remains limited but includes a randomised controlled trial evaluating a prolonged-release formulation, alongside a small number of observational studies and case reports conducted in both healthy volunteers and individuals with PKU in both slow release and prolonged release PS [18,19,21,22]. Preliminary observational findings from adult and paediatric cohorts suggest that partial substitution of conventional amino-acid PS with prolonged-release PS may enhance GI tolerance and overall acceptability [10,18,19,21,23].

In the current study, there were no notable GI concerns associated with the study product. One participant (Participant 2) reported a long-standing history of moderate to severe GI symptoms, including abdominal pain and abdominal distension, when using her existing Phe-free amino acid supplement. Following initiation of the study product, she experienced a rapid and clinically meaningful reduction in symptom severity, with complete alleviation of symptoms within the 7-day intervention period. GI symptoms are frequently under-recognised in PKU [24,25,26,27]. Although commonly reported by patients, these symptoms are rarely captured through systematic assessment in routine clinical practice or incorporated into standard quality-of-life measures. Surveys across European PKU centres have shown that although clinicians observe gastro-oesophageal reflux, flatulence, constipation, and abdominal discomfort, they are seldom formally documented during clinical follow-up, highlighting a persistent gap in the evaluation of patient-reported outcomes [28].

Improvements in GI tolerance have been reported with alternative PS formulations. For example, studies have suggested reduced GI symptom burden with CGMP-AA, tablet-based or prolonged-release amino acid products compared with traditional amino acid formulations [10,27,29]. CGMP-AA is suggested to exert prebiotic-like activity, with evidence demonstrating its capacity to modulate gut microbiota composition, characterised by reductions in potentially pathogenic taxa and enhanced short-chain fatty acids production, together with measurable improvements in intestinal inflammatory profiles [30,31,32]. Although the mechanisms explaining improved GI tolerance with prolonged-release amino acid formulations remain unclear, they are hypothesised to relate to the slower intraluminal release of amino acids and reduced gastric exposure to hyperosmolar solutions [10]. In addition, it has been proposed that cellulose-based coatings and controlled-release matrices may limit local gastric irritation and improve tolerability [15].

PS osmolality may also contribute to GI symptoms, as Phe-free amino acids PS can reach relatively high osmolality levels that have been associated with GI discomfort [33,34,35]. Data from infant studies suggest that feeds with an osmolality of 300–500 mOsm/kg are generally well tolerated, whereas higher-osmolality formulas (e.g., >600 mOsm/kg) have been linked in some reports to nausea, abdominal pain, and other adverse GI outcomes [26]. In the present study, osmolality varied substantially across baseline PS. Reconstituted powder-based amino acid PS had a wide range, approximately 750–1950 mOsm/kg, while RTD liquid formulations typically showed lower values, around 420–500 mOsm/kg. Despite these marked differences, available evidence remains inconsistent, and no robust association between osmolality and GI outcomes has been established. The slow-release microtablet formulation is not based on osmotic-driven release, and therefore osmolality is not an applicable characteristic of this product. Its release profile is governed by the hydrophilic polymer matrix and coating system, rather than by osmotic gradients.

An advantage of the study product was its micronutrient profile, incorporating additional vitamins, minerals, and trace elements. Although micronutrient status was not assessed biochemically in the present study, this feature is important given the well-documented risk of micronutrient insufficiency in individuals with PKU [36,37,38]. Such insufficiency may arise from both the inherent dietary restrictions of the low-Phe diet and variable adherence to PS. Formulations that provide a more complete nutrient profile may therefore help reduce reliance on additional supplementation and support more consistent nutritional adequacy [37]. This consideration is particularly important in paediatric and adolescent populations, where growth, bone health, and neurocognitive development substantially increase micronutrient requirements [39,40]. Moreover, concerns regarding potential micronutrient inadequacy have also been reported in individuals treated with pharmacological therapies who subsequently reduce their use of PS. This further highlights the need to ensure adequate vitamin and mineral intake across different treatment strategies in PKU [41,42,43,44].

However, an important limitation of the microtablet technology is its incompatibility with lipid-based nutrients, particularly docosahexaenoic acid (DHA) and other long-chain polyunsaturated fatty acids. These fatty acids cannot be incorporated into the tablet matrix without compromising stability or palatability. Consequently, individuals using this formulation require separate DHA supplementation to ensure adequate intake, especially given the growing evidence linking DHA status with neurocognitive outcomes, visual development, and inflammatory regulation in PKU [45,46,47,48]. Fatty acid status requires closer monitoring in patients using microtablet-based PS.

In addition, although the slow-dissolution profile of microtablets may theoretically promote a slower and more sustained release of amino acids, their specific kinetic behaviour has not yet been formally characterised. Nevertheless, several studies evaluating slow-release microtablet formulations in PKU have reported encouraging clinical outcomes, and the key findings from these investigations are summarised in Table 5. Giovannini et al. [22] investigated a slow-release formulation in paediatric patients, reporting encouraging outcomes for both metabolic control and acceptability. More recently, the PREMP study [49] demonstrated that a slow-release Phe-free formulation maintained stable Phe and Tyr concentrations over a four-month period and was associated with improved insulin sensitivity, as reflected by reductions in fasting insulin and Homeostatic Model Assessment for Insulin Resistance (HOMA-IR), together with increases in the Quantitative Insulin Sensitivity Check Index (QUICKI). Modest shifts in gut microbiota composition were also observed, including increases in Bifidobacterium, raising the possibility that slow-release formulations may exert metabolic effects extending beyond amino acid homeostasis. These findings remain preliminary, and further mechanistic studies are required to elucidate the pathways underlying these responses. In an additional clinical investigation involving individuals with PKU, slow-release amino acid formulations were associated with improved Tyr homeostasis, characterised by higher fasting and nocturnal Tyr concentrations, while maintaining stable Phe control compared with conventional amino acid-based PS [50] (Table 5).

In contrast Giarratana et al. [15] demonstrated favourable effects on plasma amino acid kinetics in an animal model using prolonged-release coated physiomimic technology. In a randomized crossover trial, Daly et al. demonstrated that a prolonged-release PS administered as the final evening dose significantly improved overnight metabolic control in children with classical PKU [51]. Compared with conventional amino acid-based PS, the prolonged-release formulation reduced early-morning blood Phe concentrations by approximately 18%, resulting in a mean Phe level of 294 μmol/L, whereas Phe concentrations increased with the conventional PS to a mean of 442 μmol/L, exceeding the recommended target range. The prolonged-release formulation was also associated with higher Tyr concentrations and a more favourable Phe/Tyr ratio [51]. In addition, a case series in pregnant women with PKU reported that prolonged-release PS was well tolerated during pregnancy and, when combined with strict dietary management, was associated with stable maternal Phe levels and favourable neonatal outcomes [21]. These findings support the concept that modified-release delivery systems may improve amino acid kinetics and promote more physiological metabolic profiles without compromising Phe stability.

Tablet-based formulations may offer individuals with PKU important psychosocial advantages, as their discreet, portable, and more socially acceptable format allows easier integration into daily routines, thereby potentially reducing the psychological burden of lifelong dietary management [52]. This aspect is particularly relevant during adolescence, a developmental period marked by heightened sensitivity to peer perception and increased vulnerability to treatment-related stigma [9,53]. Prior research has shown that visible, odorous, or socially intrusive dietary treatments in PKU can negatively affect adherence and quality of life, with some adolescents reporting embarrassment, social withdrawal, or reluctance to take PS in public settings [54,55,56,57]. In this context, the new microtablet PS represents a key strength of the intervention, as it directly addresses issues of palatability, visibility, and social acceptability that are known to limit adherence to conventional amino acid PS.

This study has several limitations that should be considered when interpreting the findings. First, the small sample size limits the generalisability of the results. As an exploratory study, it was not designed or powered to permit formal statistical inference, and the findings should therefore be regarded as preliminary. Second, the short duration of the primary intervention (7 days) precludes conclusions regarding long-term adherence, sustained GI tolerance, nutritional status, growth, or metabolic efficacy under standardised conditions. Although extended follow-up data were available for a small subgroup of participants, these observations were descriptive rather than interventional and were subject to potential confounding from variations in dietary intake, intercurrent illness, treatment adherence, and other factors encountered during routine clinical care. Third, the study was conducted in a real-world home setting, and the exact daily intake of the PS could not be independently verified. Adherence, acceptability and GI symptoms were assessed using participant or caregiver reports, which may have been influenced by reporting bias, recall bias, social desirability bias, or increased awareness associated with study participation. Fourth, participants were recruited from a single specialist metabolic centre and volunteered to evaluate this novel PS, introducing the potential for selection bias towards more motivated and treatment-engaged families. Consequently, the findings may not be representative of the wider PKU population. In addition, the study cohort included a limited range of ages and metabolic phenotypes, and the findings may not be directly applicable to younger children, adults, or individuals with poorer metabolic control. Fifth, PS prescriptions were individualised according to clinical requirements, resulting in variation in the prescribed PE dose. While this reflects routine clinical practice, it limits the ability to distinguish formulation-specific effects from those related to dose. Furthermore, participants were receiving different PS formulations before study entry, and differences in formulation composition may have influenced baseline GI symptoms, representing a potential confounding factor when interpreting changes following introduction of the microtablet. Finally, the study did not assess biomarkers of nutritional status or postprandial amino acid kinetics, which would provide important mechanistic information regarding the nutritional and metabolic effects of the formulation. Larger, multicentre, randomised controlled crossover studies with longer follow-up and direct comparison with conventional amino acid-based PS are required to confirm these preliminary findings and more fully evaluate the effects of the slow-release microtablet formulation on adherence, GI tolerance, nutritional status and metabolic outcomes.

## 5. Conclusions

This novel slow-release microtablet PS was well tolerated, acceptable, and associated with high adherence in children and adolescents with PKU. Its taste-masked, low-volume, and discreet tablet format offers practical advantages over conventional formulations, with the potential to support more consistent and sustainable dietary adherence, particularly in age groups where treatment burden and social visibility can undermine long-term adherence. The ability to distribute intake flexibly across the day may further enhance usability and integration into daily routines. In addition, the slow-release properties offer the potential for more sustained amino acid delivery and improved metabolic stability, while its comprehensive vitamin and mineral composition may help ensure nutritional adequacy and reduce the risk of micronutrient deficiencies. Taken together, these findings provide promising evidence that microtablet formulations may represent an important advancement in PKU dietary management. However, longer-term, adequately powered controlled studies are required to confirm their metabolic efficacy, evaluate their impact on nutritional status and quality of life, and determine whether the observed short-term benefits translate into sustained improvements in real-world settings.

## Figures and Tables

**Table 1:** Nutritional composition of the study product (PKU EASY Microtabs Plus—POA Pharma).

Component	Per 100 g	Per 20 g/ 25-mL
Energy	329 kcal/1428 kJ	68 kcal/286 kJ
Fat	1.85 g	0.37 g
of which saturated fat	1.55 g	0.31 g
Carbohydrates	18.0 g	3.6 g
of which sugars	0.57 g	0.11 g
Fibre	17.1 g	3.42 g
Protein	54.0 g	10.8 g
Protein equivalent *	50.0 g	10.0 g
Salt	1.32 g	0.26 g
	**Amino Acids**	
L-Alanine	2.17 g	0.43 g
L-Arginine	3.46 g	0.69 g
L-Aspartate	5.49 g	1.10 g
L-Cystine	1.42 g	0.28 g
Glycine	5.43 g	1.09 g
L-Glutamine	4.26 g	0.85 g
L-Histidine	2.17 g	0.43 g
L-Isoleucine	3.75 g	0.75 g
L-Leucine	5.85 g	1.17 g
L-Lysine	3.89 g	0.78 g
L-Methionine	1.0 g	0.20 g
L-Proline	3.92 g	0.78 g
L-Serine	2.42 g	0.48 g
L-Threonine	3.75 g	0.75 g
L-Tryptophan	1.17 g	0.23 g
L-Tyrosine	5.50 g	1.10 g
L-Valine	4.34 g	0.87 g
Phenylalanine	Absent	Absent
	**Vitamins**	
Vitamin A	667 μg	133 μg
Vitamin D	8.4 μg	1.67 μg
Vitamin E	7.5 mg	1.51 mg
Vitamin K (as K2)	58.3 μg	11.7 μg
Vitamin C	41.7 mg	8.33 mg
Thiamin	1.0 mg	0.20 mg
Riboflavin	1.2 mg	0.23 mg
Niacin	16.7 mg	3.33 mg
Vitamin B6	1.3 mg	0.27 mg
Folic Acid	585 μg	117 μg
Vitamin B12	4.2 μg	0.83 μg
Biotin	20.8 μg	4.15 μg
Pantothenic acid	4.2 mg	0.83 mg
	**Minerals**	
Sodium	528 mg	106 mg
Potassium	161 mg	32 mg
Calcium	667 mg	133 mg
Phosphorus	643 mg	129 mg
Magnesium	250 mg	50 mg
Iron	12.5 mg	2.5 mg
Zinc	9.2 mg	1.8 mg
Copper	1.25 mg	0.25 mg
Manganese	1.25 mg	0.25 mg
Selenium	63 μg	12.6 μg
Chromium	25.3 μg	5.1 μg
Molybdenum	58.3 μg	11.7 μg
Iodine	126 μg	25.2 μg
	**Other Nutrients**	
Taurine	0.085 g	0.017 g
L-Carnitine	0.057 g	0.011 g

* 1 g of protein equivalent = 1.2 g of amino acids. The protein content is provided by the amino acids. Ingredients: bulking agents: cellulose, hydroxypropyl methyl cellulose, hydroxypropyl cellulose; stabilizers: sodium alginate, ethyl cellulose, ammonium hydroxide; L-lysine- L-aspartate, L-leucine, L-tyrosine, glycine, L-arginine-L-aspartate, L-valine, L-glutamine, L-proline, L-isoleucine, L-threonine, calcium salts of orthophosphoric acid, glazing agents: calcium carbonate, hydroxypropyl-methylcellulose, isomalt, medium chain triglycerides; L-serine, L-alanine, L-histidine, anticaking agents: magnesium salt of fatty acids, fatty acids; L-cystine, L-tryptophan, magnesium carbonate, L-methionine, acidity regulator: citric acid; L-arginine, potassium salts of orthophosphoric acid, medium chain triglycerides, L-carnitine L-tartrate, taurine, ferrous citrate, L-ascorbic acid (vitamin C), menaquinone (Vitamin K), zinc citrate, DL-alpha-tocopherol acetate (Vitamin E), nicotinamide (Niacin), sodium molybdate, colours: iron oxides and hydroxides; manganese gluconate, D-pantothenate calcium (pantothenic acid), retinyl acetate (Vitamin A), cyanocobalamin (Vitamin B12), cholecalciferol (vitamin D), copper citrate, pyridoxine hydrochloride (Vitamin B6), thiamine hydrochloride (Vitamin B1), riboflavin (Vitamin B2), pteroylmonoglutamic acid (Folic acid), chromium picolinate, potassium iodide, sodium selenite, biotin.

**Table 2:** Individual PS use and daily protein equivalent intake at baseline (T0) and day 7 (T1).

Subject	NP (Exch)	PE (g/Day)	Type and Doses of the Usual PS	Adherence Amount; Dose
1	3	80	RTD-AA, 4 doses/d	0; 0
2	3	80	RTD-AA, 4 doses/d	1; 1
3	3	80	RTD-AA, 4 doses/d	1; 1
4	4	60	RTD-AA, 3 doses/d	0; 0
5	4	60	RTD-AA, 3 doses/d	0; 0
6	13	70	AA Powder with water, 3.5 doses/d	0; 0
7	7.5	80	AA Tablets with water, 4 doses/d	0; 0
8	7	80	CGMP-AA Powder with LP milk, 3 doses/d and RTD-AA, 1 dose/d	0; 0
9	3.5	60	RTD-AA, 4 doses/d	0; 0
10	20	80	AA Microtabs *, 4 doses/d	1; 1
**Subject**	**NP** **(Exch)**	**PE (g/day)**	**Prescribed doses of the study PS**	**Adherence** **Amount; Dose**
1	3	60 + 20	RTD-AA, 3 doses/d + study PS, 1 dose/d (2 × 25 mL cups)	0; 0
2	3	60 + 20	RTD-AA, 3 doses/d + study PS, 1 dose/d (2 × 25 mL cups)	0; 0
3	3	60 + 20	RTD-AA, 3 doses/d + study PS, 1 dose/d (2 × 25 mL cups)	1; 1
4	4	40 + 20	RTD-AA, 2 doses/d + study PS, 1 dose/d (2 × 25 mL cups)	0; 0
5	4	40 + 20	RTD-AA, 2 doses/d + study PS, 1 dose/d (2 × 25 mL cups)	0; 0
6	13	50 + 20	AA Powder with water, 2.5 doses/d + study PS, 1 dose/d (2 × 25 mL cups)	0; 0
7	7.5	60 + 20	AA Tablets with water, 3 doses/d + study PS, 1 dose/d (2 × 25 mL cups)	0; 0
8	7	60 + 20	CGMP-AA Powder with LP milk, 3 doses/d + study PS, 1 dose/d (2 × 25 mL cups)	0; 0
9	3.5	60 + 10	RTD-AA, 4 doses/d + study PS, 1 dose/d (1 × 25 mL cups)	0; 0
10	20	80	Study PS, 4 doses/d (8 × 25 mL cups)	1; 1

* Participant 10 was already using a microtablet PS, though not the study formulation. Full dose taken: 0 = Always, 1 = Mostly, 2 = Occasionally, 3 = Rarely, 4 = Very Rarely, 5 = Never. Amount taken: 0 = All, 1 = 75%, 2 = 50%, 3 = 25%, 4 = None. Abbreviations: PS = Protein Substitute; NP = Natural Protein; Exch = Protein Exchanges; PE = Protein Equivalent; RTD-AA = Ready To Drink Amino Acid based PS; AA = Amino Acids; CGMP-AA = Casein Glycomacropeptide supplemented with Amino Acids; LP = Low Protein.

**Table 3:** Comparison of current PS with microtablet formulation (median; range).

Acceptability	Current PS Median (Range)	Study PS Median (Range)
Appearance	2 (0–3)	2 (0–3)
Smell	2 (1–4)	2 (0–4)
Taste	1.5 (0–3)	2 (0–3)
Texture	2 (0–4)	2 (1–4)
Ease of mixing	1.5 (1–2) *	N/A
Ease of taking	1 (0–3)	1.5 (0–3)
Aftertaste	3 (1–3)	2 (0–4)
Overall	2 (0–3)	2 (0–3)

* Only two participants were taking PS that require mixing. N/A indicates not applicable, as the microtablet formulation does not require mixing. Acceptability was scored from *Great* (0) to *Terrible* (4), with an additional option of *Not applicable*. Abbreviations: PS = Protein Substitute.

**Table 4:** Individual mean Phe and Tyr during the 28-day post-intervention follow-up period.

Subject	Age (Years, Months)	Number of DBS	Mean Phe µmol/L	Reference Range (µmol/L)	% of DBS Within the Range	Mean Tyr (µmol/L)
2	11.8	3	423	120–360	33.3%	123
6	12.6	3	300	120–600	100.0%	40
8	14.4	4	467	120–600	100.0%	75
10	15.0	2	380	120–600	100.0%	136

Abbreviations: DBS = Dried Blood Spot; Phe = Phenylalanine; Tyr = Tyrosine.

**Table 5:** Clinical studies investigating the same slow-release amino acid formulation.

Reference	Study Product	Study Design	Population	Intervention	Main Findings
Giovannini et al., 2014 [22]	Slow release Phe-free PS	Single-centre randomized controlled trial	Children with PKU (mild n = 40; classical n = 15). Controls: Children with HPA (n = 60) and unaffected subjects (n = 60)	Administration of the slow release PS (3–4 daily doses), or standard amino acid PS, for 30 days	Albumin and retinol-binding protein remained within the reference range. Protein insufficiency (transthyretin < 20 mg/dL) was similar between groups. Transthyretin increased in the test group (+1.6 mg/dL), with minimal change in the standard PS group (+0.2 mg/dL). A greater reduction in Phe was observed in the test group (mean change −1.75 mg/dL) compared with the control group (−0.40 mg/dL). Adherence was similar between groups (~81–82%). Acceptability was good, although swallowing difficulty was more frequently reported with the test PS.
Porta et al., 2020 [50]	Slow release Phe-free PS	Single-centre interventional study	Patients with PKU/HPA: n = 114 (classic or mild PKU: n = 52; HPA: n = 62). Controls: healthy subjects (n = 4, for Tyr loading test)	Switch from usual Phe-free PS to a Phe-free slow-release PS (0.9 ± 0.1 g/kg/day in 3 daily doses) for 6 months	Tyr concentrations were significantly lower in PKU compared to HPA (*p* < 0.01). The Tyr loading test showed a normal metabolic response in PKU. Slow-release PS was associated with higher morning fasting and nocturnal Tyr concentrations compared with traditional therapy (*p* < 0.019. At follow-up, normalization of fasting Tyr levels under slow-release PS (*p* < 0.01) and no significant change in Phe control (*p* = 0.19).
Tosi et al., 2025 [49]	Slow release Phe-free PS	Two-centre interventional study	Patients with PKU: n = 13 (classic PKU: n = 9; other forms not specified). Controls: not included	Substitution of ≥50% of usual protein intake with Phe-free slow-release PS for 4 months	No significant changes were observed in plasma Phe, Tyr, or Phe/Tyr ratio. Significant improvements were found in fasting insulin, HOMA-IR, and QUICKI, while glucose showed a non-significant decreasing trend. Gut microbiota alpha and beta diversity did not change significantly, although minor trends in increased richness and modest shifts in selected genera were observed. Faecal fatty acids showed non-significant increases in butyrate and other SCFAs.

Abbreviations: PS = Protein Substitute; PKU = Phenylketonuria; HPA = hyperphenylalaninemia; Phe = Phenylalanine; Tyr = Tyrosine; HOMA-IR: Homeostatic Model Assessment of Insulin Resistance; QUICKI = Quantitative Insulin Sensitivity Check Index; SCFAs = Short Chain Fatty Acids.

## Data Availability

The original contributions presented in this study are included in the article/Supplementary Material. Further inquiries can be directed to the corresponding author.

## Supplementary Materials

The following supporting information can be downloaded at: https://www.mdpi.com/article/10.3390/nu18142375/s1, Table S1. Demographic data for children with PKU at T0 (baseline). Table S2. Anthropometric data for children with PKU at T0 (baseline) and T1 (day 7).
