# Supplementary material for: Use of a Slow-Release Phenylalanine-Free Microtablet Protein Substitute in Children and Adolescents with Phenylketonuria: An Observational Pilot Study
Source: Nutrients. 2026 Jul 21;18(14):2375. doi: 10.3390/nu18142375 (PMC13416016; doi:10.3390/nu18142375)
Supplement: Supplementary file 1 [file nutrients-18-02375-s001.zip › nutrients-4397686-supplementary.pdf]

Supplementary Table S1. Demographic data for children with PKU at T0 (baseline).

Subject	Gender (F, M)	Age (years, months)	Ethnicity
**1**	M	14.7	White British
**2**	F	11.8	White - other
**3**	M	10.7	White - other
**4**	F	16.6	British Pakistani
**5**	F	16.6	British Pakistani
**6**	F	12.6	White British
**7**	M	17.5	White British
**8**	M	14.4	White - other
**9**	M	5.1	White - other
**10**	F	15.0	White British

Supplementary Table S2. Anthropometric data for children with PKU at T0 (baseline) and T1 (day 7).

			**T0**			
**Subject**	**Height (cm)**	**z-score**	**Weight (kg)**	**z-score**	**BMI**	**z-score**
**1**	155.2	-1.44	48.8	-0.51	20.3	0.47
**2**	149.0	0.03	44.7	0.63	20.1	0.81
**3**	143.1	0.18	37.7	0.64	18.4	0.78
**4**	158.9	-0.76	83.2	2.44	33	2.78
**5**	157.8	-0.94	80.6	2.26	32.4	2.7
**6**	161.3	1.14	68.7	2.36	26.4	2.2
**7**	180.2	0.5	67.6	0.2	20.8	0.01
**8**	181.0	1.85	83.0	2.38	25.3	1.96
**9**	120.5	2.25	26.8	2.62	18.5	1.92
**10**	160.0	-0.33	48.0	-0.70	18.75	-0.5
			**T1**			
**Subject**	**Height (cm)**	**z-score**	**Weight (kg)**	**z-score**	**BMI**	**z-score**
**1**	155.3	-1.44	48.1	-0.6	19.9	0.35
**2**	149.1	0.03	45.4	0.7	20.4	0.9
**3**	143.1	0.16	37.8	0.63	18.4	0.78
**4**	158.9	-0.76	83.3	2.45	33	2.79
**5**	157.9	-0.92	80.4	2.24	32.2	2.68
**6**	161.3	1.12	68.2	2.32	26.2	2.16
**7**	180.2	0.49	67.9	0.22	20.9	0.04
**8**	181.4	1.89	83.0	2.37	25.2	1.93
**9**	120.5	2.21	26.7	2.56	18.4	1.87
**10**	160.1	-0.32	48.1	-0.7	18.8	-0.49
